# Investigating the monetary policy risk channel based on the dynamic stochastic general equilibrium model: Empirical evidence from Iran

**DOI:** 10.1371/journal.pone.0291934

**Published:** 2023-10-05

**Authors:** Pejman Peykani, Mostafa Sargolzaei, Amir Takaloo, Negin Sanadgol

**Affiliations:** 1 School of Industrial Engineering, Iran University of Science and Technology, Tehran, Iran; 2 Department of Finance and Banking, Faculty of Management and Accounting, Allameh Tabataba’i University, Tehran, Iran; 3 School of Management, Economics and Progress Engineering, Iran University of Science and Technology, Tehran, Iran; Palestine Economic Policy Research Institute, STATE OF PALESTINE

## Abstract

Investigating the credit channel and monetary policy risk channel in Iran’s economy is the aim of this article. According to empirical studies, expansionary monetary policy increases the risk of banks, and on the other hand, the risk of banks affects economic activities and price levels. In order to investigate the mechanism of the credit channel and the risk channel (as a new channel), the effect of monetary policy on real variables and price levels in Iran’s economy, the Dynamic Stochastic General Equilibrium (DSGE) model has been used by entering the information of the banking system and considering moral hazard and adverse choices. The obtained results show that there is a credit channel and a monetary policy risk channel for Iran’s economy, and the expansionary monetary policy shock causes output, inflation, private sector consumption, investment, net worth in the economy and lending to increase. Also, when a credit shock occurs, with the increase in banks’ lending power, production, private sector consumption, investment, net worth and total lending increase and the inflation level decreases. Also, by applying the risk shock caused by the increase in inflation and the decrease in consumption and investment, the volume of lending increases and the level of production does not change much.

## 1. Introduction

The importance of economic policies in economic development, on the one hand, and the effects of wrong policies and their destructive power on the other hand, make attention to how economic policies are applied and how they work always be the main topic of economists’ analysis [1–4]. From the point of view of economic scientists, the effect of applied policies is different and how and by changing which variables and markets affect the target variables of the policy [5–8].

The evolution of macroeconomic perspectives on the monetary transmission mechanism shows that the effects of monetary policy on real output and inflation have changed dramatically in recent decades [9–19]. The study of monetary policy is important not only in terms of its impact on economic variables, but also in terms of helping monetary decision makers and policymakers to evaluate economic policies more accurately [20–26]. Considering the growing importance of financial markets both at the national and international levels and the effectiveness of such markets in terms of monetary policies, the effectiveness of monetary policies through its various mechanisms has been researched more than before. For example, the global financial crisis in recent years and the measures taken by the Central Bank of Europe and the United States to solve it, or the use of quantitative easing policies, showed that the importance of monetary policies and its effectiveness on all types of markets and the realization of economic growth and inflation control is undeniable [27–33]. Secondly, it is necessary to pay attention to the mechanisms and channels of monetary transmission in order to make policies more effective [34–38]. Obviously, a correct assessment will not be possible without a proper understanding of the mechanisms by which monetary policy will affect economic variables. One of the important advances in the field of monetary transmission mechanism studies in recent years has been the identification of financial market conditions and their progress as one of the environmental factors affecting the monetary transmission mechanism and the effect of fiscal policy is different in different countries [39].

Monetary policy transmission refers to the effect of monetary policy on important macroeconomic variables such as production and inflation. In this regard, the monetary policy transmission mechanism deals with the effect of this policy and explains the channels through which monetary policy affects inflation and the real sector of the economy. In this regard, in addition to the channels of interest rate, exchange rate, asset price, credit channel and expectations channel, in the last few years and after the financial crisis of 2008, a new channel called the risk channel has received attention [19, 38, 40–42].

The risk channel refers to the fact that monetary policy can be effective on real economic activities by influencing the risk-taking of monetary and financial sector actors, especially banks. The experience of the financial crisis of 2008 showed that following an expansionary monetary policy with increased risk-taking, banks offered risky loans that this issue caused an increase in the debt-to-asset ratio of banks and finally led to financial instability and crisis. Therefore, various researches have been conducted all over the world in the field of monetary policy risk channel [43, 44]. Despite the risk channel, the expansionary monetary policy leads to an increase in banks’ risk-taking and the supply of risky loans. As a result of these conditions, the strength of the banking system is endangered and the financial system may face instability [45, 46].

The growth of real economic activities depends on financing investment projects. The banking system plays an important role in financing all over the world, and accordingly, in Iran’s economy, banks play an important role in financing investment projects [47]. From this point of view, the monetary policy risk channel has a negative effect on the real activities of the economy by disrupting the strength of the banking system, because one of the negative consequences of risk-taking is the supply of risky loans and the increase in the probability of non-repayment of the loan, and as a result, reducing the ability of banks to lend to Investment projects [47–49].

According to statistics published by the Central Bank of Iran, during the years 2014 to 2021, the ratio of non-current facilities to total facilities paid by banks in Iran was 11.4, 17.6, 18.2, 13.8, 15.1, 14.7, 14.1 and 12.1 percent, respectively. As can be seen, in recent years, the ratio of non-current facilities to total payment facilities in Iran’s banking system is more than 18%, while the normal limit of the ratio of non-current facilities to total payment facilities is less than 18%, as one of the indicators Financial stability introduced by the International Monetary Fund. Therefore, the increase in the amount of non-current facilities in Iran makes the hypothesis of the existence of a risk channel in the banking system of Iran stronger.

If the hypothesis of the existence of bank risk channel in Iran is accepted, this factor can be considered as one of the factors of increasing non-current facilities in Iran’s banking system. As stated, following the expansionary monetary policy, banks offer risky loans by increasing risk-taking, and this issue has increased the probability of default and non-current facilities. On the other hand, according to the banking risk channel in Iran, the central bank should reconsider its optimal policy design, because with the existence of the risk channel, the parameters related to the objective function of the central bank and the optimal monetary policy change. In other words, the central bank can minimize the negative effects of this channel on Iran’s economy by considering the losses caused by the increase in bank risk-taking in its loss function and designing the optimal policy under these conditions.

Considering the importance of the bank’s risk channel in the design of monetary policy and the connection of this channel with the financial stability and strength of the banking system, the main purpose of this research is to investigate the credit channel and risk channel of monetary policy. Dynamic Stochastic General Equilibrium (DSGE) model has been used to investigate the mechanism of credit channel and risk channel (as a new channel) and the effect of monetary policy on real variables and price levels in Iran’s economy.

The rest of this article is organized as follows. In section 2, researches that have been done on this issue in the past are mentioned. In section 3, all the equations of the proposed model are stated, which were necessary to achieve the goal of this article. Also, in section 4, the results obtained from the proposed model are analyzed based on the information used. Finally, finally, in section 5, the final conclusions are stated and the suggestions for the future to expand the concept are introduced.

## 2. Literature review

In this section, an overview of applied research on Monetary Policy is presented, which has affected the economy of different countries using different models.

According to the credit channel theory, monetary policy not only affects the general level of interest rates, but also affects the cost of external financing. This transmission at the cost of external financing can work better in the timing and combination of monetary policy effects. Based on this, two mechanisms of the effect of monetary policy on the cost of external financing in credit markets have been explained by Bernanke and Gretler [50], which are balance sheet channel, which is sometimes referred to as net worth, and bank loan channel. In the balance sheet channel, the potential effect of monetary policy on borrowers’ balance sheets and their income is investigated. While in the bank loan channel, the focus is more on the effect of monetary policy on the supply of loans by deposit-taking institutions such as banks. The balance sheet channel is based on a theory that attributes the cost of external financing to borrowers to their financial position or net worth. Net worth is the sum of cash assets and mortgage bonds of borrowers that can be sold in the market. Despite the larger net worth, the financial situation of the borrowers is improved and enables the borrower to reduce the cost of external financing while increasing his potential bargaining power. The reason for this is either due to the increase in the share of internal financial provision from the total investment, or because of the provision of more mortgages or bonds for guarantee. Transmission and change in the central bank’s policy not only affects the market interest rate, but also directly and indirectly affects the financial conditions of the borrowers. Contractionary monetary policy and increase in interest rates are generally accompanied by a decrease in the price of assets, which reduces the price and value of the borrower’s collateral. In this way, the loan received by the borrowers is limited by the value of the assets that they can use as collateral. As interest rates increase and asset prices decrease, the market value of collateral also decreases. This decrease in the value of collateral forces some enterprises to reduce their investment expenses because their ability to borrow decreases.

In addition to the effect of monetary policy on the borrower’s balance sheet, monetary policy has an effect on the cost of external financing through the transmission of commercial banks’ credit supply, which is known as bank loan channel. The bank loan channel emphasizes the special nature of bank credit and the role of banks in the financial structure of the economy. In the bank loan approach, banks play an important role in the transmission of monetary policy to the real economy, and bank loans are an important substitute for financial instruments. If economic policies affect banks’ reserves, it creates adjustments in interest rates and balance sheet elements of the banking sector. Effects on banking sector reserves and interest rates affect the supply of bank credit and affect the asset side of the balance sheet. If the banks can’t solve the decrease in reserves by adjusting the stock assets or increasing the capital, then the supply of bank loans will decrease. Walsh [51] has proposed that if bank borrowers do not have suitable substitutes to access capital, the change in bank loan supply may have an independent effect on the total cost. As the supply of bank loans decreases, borrowers will incur costs related to searching for new lenders. Since many banks are facing the problem of obtaining information and most of the borrowers are also dependent on bank loans. Therefore, the decrease in the supply of bank credit compared to other credits leads to an increase in the cost of external financing and a decrease in real economic activities.

In recent studies about the monetary transmission mechanism and the role of monetary policies in the economy, the role of bank credits and the special importance of their risk have been discussed, and a new approach about the effects of monetary policy on the real variables of the economy through the risk channel has been emphasized. Brio and Zhu [52], Angeloni et al. [53] and also Bruno and Shin [54] have tried to introduce a new relationship between the real sector and the financial sector of the economy by introducing a new channel of the monetary mechanism. In fact, the monetary policy can affect the real variables by influencing the banks’ risk. The main hypothesis that is investigated in the channel of risk monetary transmission mechanism is that by reducing the interest rate in a long period, it causes an increase in bank loans to risky customers and this risk increases the credit portfolio of the bank, which will have an effect on the real variables as well as the price level with the increase in bank credit defaults.

To better understand how the risk channel is effective, we introduce two main approaches. Rajan [55] has explained that the first approach occurs with a change in the degree of risk of intermediate assets. In reducing or stabilizing interest rates, bank asset managers have the incentive to invest in riskier conditions to earn more profit. Adrien and Shin [56] have also stated that the second approach occurs through the capital structure of banks, so that an expansionary monetary policy can affect the composition of bank liabilities.

Aysun [57] has investigated the effect of economic shocks through the credit channel on lending to small and large banks. He has come to the conclusion that the lending of large banks is more sensitive to economic shocks than small banks, and the balance sheet of larger borrowers is more sensitive to economic shocks than smaller borrowers. At the end of the article, it is concluded that Shocks are transmitted to the real economy mainly through large bank loans and large borrowers’ balance sheets using the DSGE model.

Yagihashi [58] have investigated the effect of credit market friction on the increase in the cost of monetary policy. To achieve this goal, the DSGE model and the credit channel model defined by Bernanke and Gretler [50] have been used. The obtained results show that the credit channel model is a policy guidance tool. In this article, it is stated that credit market friction should be considered in the model, otherwise the opportunity cost of using an inappropriate model is greater than the obtained results.

Taguchi and Gunbileg [59] have tried to analyze the effects of the monetary policy of the Bank of Mongolia and the policy in which inflation is targeted at a certain rate using the country’s economic data and with the help of two different economic models, one of which is DSGE model. One of the issues in this article is to determine how closely monetary policy in this country is in line with the Taylor principle. The results of the study show that both models confirm that the policies are in line with the Taylor principle, but the power of these policies is less against inflation than other neighboring countries.

De Jesus et al. [60] have tried to investigate the effect of monetary policy shock on various macroeconomic parameters assuming that the economy has financial constraints and have used the DSGE model to analyze this issue using Brazilian macroeconomic data. As mentioned, it has been assumed that the government is limited in its spending, but in this study, three different models with different assumptions have been considered, one of which is based on the fact that there are no restrictions And another model in which it is assumed that the government is facing a very severe limit on its spending, and the last model in which it is assumed that there is a financial constraint for the government but it is not very strict. The results show that the responses of variables to economic shocks in the form of two models without limitation and normal limitation are very slightly different, but the responses based on the model with severe limitation are significantly different. Another finding of this study is that the level of well-being in a society where the government faces low-intensity spending limits is higher than when it struggles with severe limitations.

In a research, Wang [31] has stated that if monetary policymakers want to reduce inflation at the macroeconomic level, they should use quantitative easing policy. The authors of this study have presented a DSGE model using the economic data of Japan in 2013 based on government bonds with different maturities and they have tried to analyze the relationship between the behavior of the Bank of Japan and the implementation of a policy of quantitative easing with inflation and interest rates. The results show that there is a significant relationship between these parameters in that the increase in asset purchases by the central bank increases inflation in the long run and is inversely related to interest rates. Another important result is that the duration of the effect of the easing policies on inflation and interest rates has been slightly different, meaning that the effect of this policy on inflation has been established in the short term and then disappeared after a while. But its impact on interest rates has been long-term.

In a research, Luo et al. [30] have tried to find out what effect electronic money has on the monetary policies of different economies and in this way they have used the DSGE model in the New Keynesian framework. The noteworthy point is that in the presented model, three sectors of household, bank and central bank have been analyzed. To investigate this effect, various variables have been used in the model such as savings, loans, output and interest rates. The obtained results show that the relationship between electronic money and savings and the relationship between electronic money and loans is asymmetric and has a large deviation. Also, the relationship between electronic money and interest rate is inverse. At the end of this article, there are suggestions for correct monetary policy making by economic managers.

Nguyen et al. [61] have sought to analyze the monetary policies affected by Covid-19, one of the biggest challenges that the entire world has faced in recent years. As it is evident, different countries have been struggling with this disease for many years and their economy suffered a severe recession. The purpose of this article was to investigate the impact of this epidemic on the monetary policies adopted in the Vietnamese economy. To achieve this goal, the DSGE model has been used and the obtained results show that a 1.5% increase in the outbreak of this disease has caused a decrease of about 1% in the output gap in the first quarter and then an increase. Other variables such as interest rates, inflation, and exchange rate changes have been used, which show their decrease.

Zhang et al. [62] have tried to analyze the transmission mechanism and the impact of oil price fluctuations in the Chinese economy. To achieve this goal, they used the economic information of the Chinese economy between 1996 and 2019 and the DSGE model. The obtained results show that oil price fluctuations have a significant effect on output. From the results, it is evident that the decrease in oil price leads to the growth of output due to the decrease in cost. When the price of oil grows, the economic demand changes and the output increases again, and the exchange rate channel is one of the ways that can transfer the demand for oil and reduce the total demand, which consequently reduces the output. At the end of this article, there are suggestions about monetary policies to deal with the fluctuations of economic variables, including oil prices.

The review of past studies clearly shows that the void of this research is felt, in the sense that due to the complexity of the DSGE model, few people have investigated various economic issues through this model. It is also understandable that investigating the monetary policy risk channel based on the DSGE model has been done for the first time in Iran.

## 3. Methodology

In this section, we intend to express the equations used in the proposed model and the different conditions of the model.

**Fig 1:**
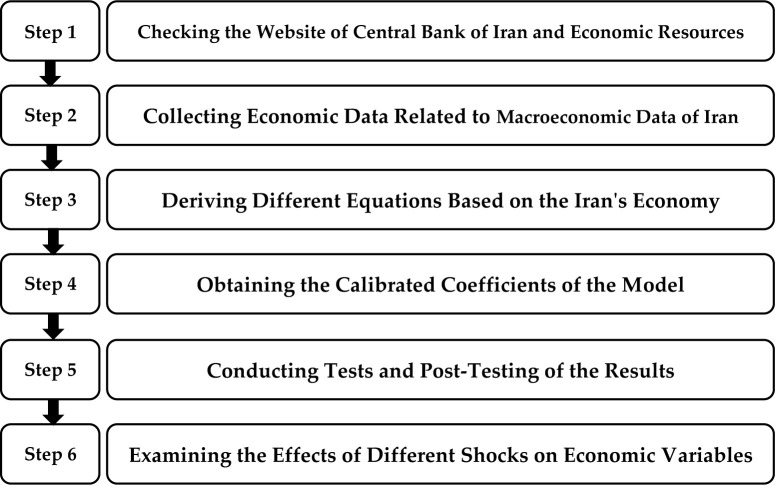
The schematic summary of all steps in the proposed DSGE model.

Fig 1 shows a schematic of the proposed method in this research.

**Fig 2:**
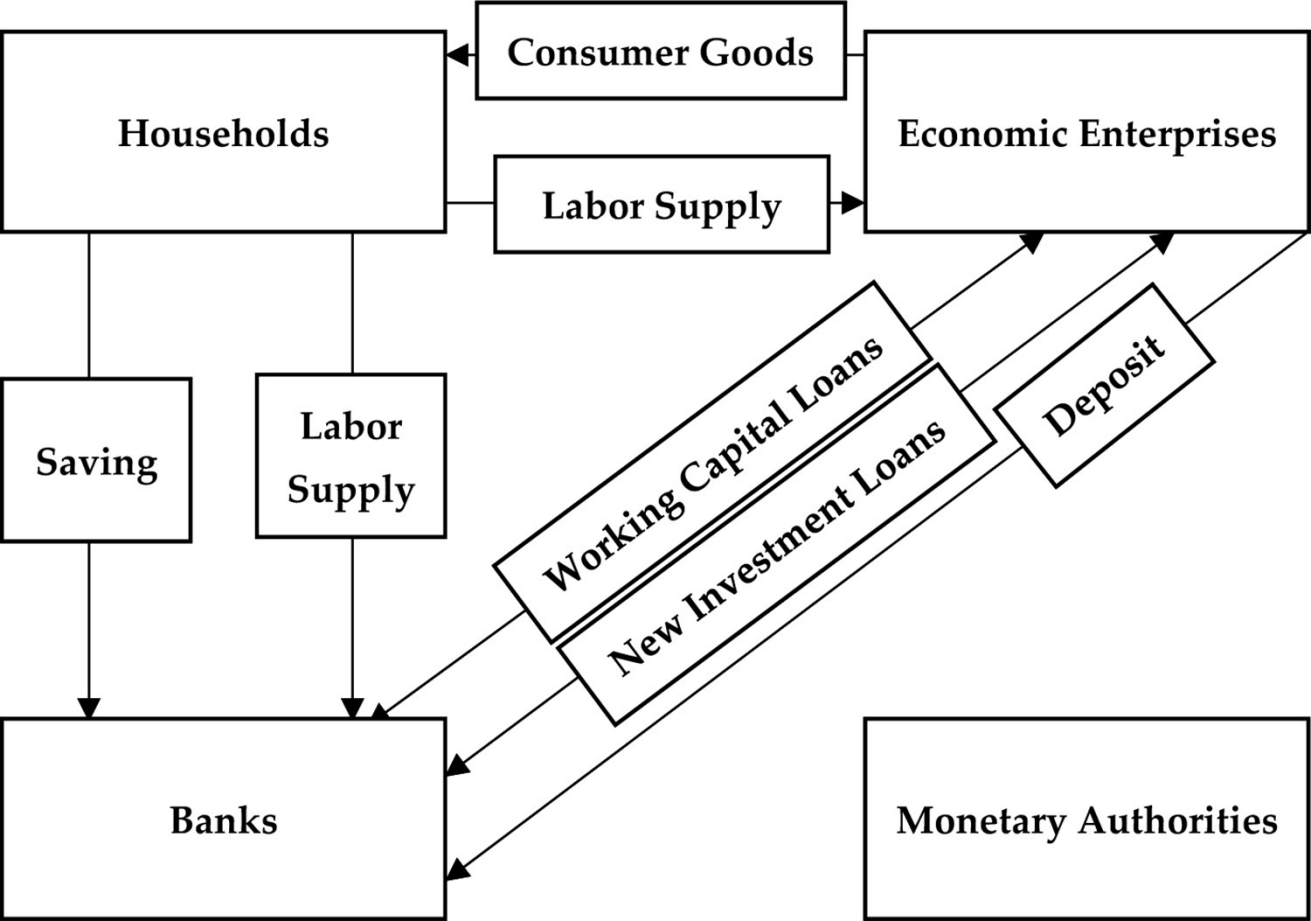
The relationship between different parts in the presented model.

The model presented in this research is the modified model of Christiano et al. [63–65], along with adding the part of the financial contract between the employer and the bank from the model of Bernanke et al. [66]. The model presented in this research consists of 4 general sectors: household, enterprise (enterprises producing intermediate goods, enterprises producing final and capital goods and trust enterprise), bank and monetary authority. Before introducing each of the sections, the relationship between these sections can be briefly stated as follows:

Households have raw materials and labor and enterprises have homogeneous capital that they supply in the market of production factors. Also, households have powerful money that they either keep in cash or deposit in banks. Households will not receive interest by keeping cash and will use cash for exchange services. But on the other hand, interest is paid to bank deposits, and these deposits will be used in the banking system for the purpose of providing liquidity services to enterprises. Banks use household deposits to provide loans to enterprises. It is also worth mentioning that enterprises producing intermediate goods need bank loans to finance salaries and wages, capital lease and new investment. Enterprises and banks need labor and capital to use them for the production process and liquidity services, respectively. The relationship between different parts can be shown in Fig 2.

An important point that should be mentioned here is that the common and conventional approach to investigate monetary policy channels is to use the DSGE model, and the creation of DSGE structures is one of the requirements of the central banks of different countries. Another important point that should be mentioned is that the DSGE model examines all economic agents, including producers, government, banks, etc., and in this model, we can analyze their functions [58–62, 67, 68].

### 3.1 Economic enterprises

In the goods producers section, there are two types of producers named final goods producers and intermediate goods producers. The final good is produced by enterprises with the following process in a competitive market and is denoted by *Y*_*t*_.

$$Y_{t}={\left[\int_{0}^{1}Y_{jt}^{\frac{1}{\lambda_{f.t}}}dj\right]}^{\lambda_{f.t}}.0<\lambda_{f.t}<\infty$$ (1)

where *Y*_*jt*_ represents the jth intermediate good at time t, *λ*_*f*.*t*_ represents the technology shock, and j is normalized between zero and one. If we call *P*_*t*_ and *P*_*jt*_ the price of the final good and the price of the intermediate good respectively, then the enterprise seeks to maximize profit according to specific and determined prices. Regarding the intermediate goods j used in Eq 1, it should be said that it will be in a monopoly market with the following production process:

$$Y_{jt}=\varepsilon_{t}K_{jt}^{\alpha }{\left(z_{t}l_{jt}\right)}^{1-\alpha }\Phi z_{t}^{*}.0<\alpha <1$$ (2)

where *K*_*jt*_ and *l*_*jt*_ represent capital services and homogeneous labor respectively, and Φ represents the non-negative parameter of fixed production costs, and *ε*_*t*_ represents a technology shock for the producer of intermediate goods. Also *z*_*t*_ represents the stable component of technology, which is expressed as the following time series:

$$z_{t}=\mu_{z.t}z_{t-1}$$ (3)

While in Eq 3, *μ*_*z*.*t*_ is a random process. On the other hand, labor supply by households is expressed in the following equation:

$$l_{t}={\left[\int_{0}^{1}h_{j.t}^{\frac{1}{\lambda_{\omega }}}dj\right]}^{\lambda_{\omega }}.1\leq \lambda_{\omega }$$ (4)

We will describe how to determine *h*_*j*.*t*_ in detail in the household section. Regarding the changes in the price, we behave according to the Calvo model [2], so that in each period t, a percentage of the intermediate goods enterprises shown as (1-*ξ*_*p*_) can adjust their price by optimizing their profit maximization. to change in that case, for enterprises that cannot determine the price optimally in period t, then it will be determined as follows:

$$P_{i.t}={\tilde{\pi }}_{t}P_{i.t},\;{\tilde{\pi }}_{t}={\left(\pi_{t}^{target}\right)}^{l}{\left(\pi_{t-1}\right)}^{1-l},\;\pi_{t-1}=\frac{P_{t-1}}{P_{t-2}}$$ (5)

We also have that $\pi_{t}^{target}$ also represents the target inflation in the function of the monetary policy maker, which we will explain more about in the monetary policy maker section. But for the enterprise that can determine the price optimally, then we have:

$$P_{i.t}={\tilde{P}}_{t}$$ (6)

### 3.2 Trust enterprises

It is assumed that there are many trust enterprises. A trust enterprise has a net worth *N*_*t*+1_ at the end of period t. At the end of period t, the trust enterprise combines its net worth with the bank loan it receives from the banks so that it can pay m to the enterprises producing intermediate goods for the purpose of new investment ${\bar{K}}_{t+1}$. The trust enterprise faces a risk shock, *ω*. so that the purchased capital ${\bar{K}}_{t+1}$ becomes ${\bar{K}}_{t+1}\omega$ so that m has a lognormal distribution and is a random variable among all trust enterprises whose cumulative distribution function is shown as *F*_*t*_(*ω*) and has a mean of $\mu_{\bar{\omega }}$ and a standard deviation of *σ*_*t*_. In a situation where we are in a long-term stable state, the parameter $\mu_{\bar{\omega }}$ will be such that *Eω* is equal to one and *σ*_*t*_ has a random process, which we call risk shock in the following model statement. The random variable *ω* is visible to the trust enterprise, but it will be visible to the bank if it pays the monitoring fee for the trust enterprise. After observing the shocks in the period t+1, the trust enterprise determines the capital utilization rate, denoted by *u*_*t*+1_, and then rents capital services to producers of intermediate goods in a competitive market. We denote the rental rate of a capital unit by ${\tilde{r}}_{t+1}^{k}$. In order to determine the capital utilization rate, the trust enterprise uses the cost function:

$$P_{t+1}\psi^{-(t+1)}a(u_{t+1})\omega {\bar{K}}_{t+1}u=1,\;a(1)=0,\;a^{\prime }(u)=r^{k},\;a^{\prime \prime }(u)=\sigma_{a}r^{k},\sigma_{a}=\frac{a^{\prime \prime }(u)}{a^{\prime }(u)}$$ (7)

where *r*^*k*^ represents the capital rental rate in a long-term stable state and *σ*_*a*_ is also a parameter that represents the degree of convexity of the trust enterprise cost function.

After determining the capital utilization rate and the income from the capital rental rate (net capital utilization income), trust enterprise sells the undepreciated capital to the producer of the final product at price $Q_{\bar{k}.t+1}$. Therefore, the total receipt of trust enterprise in period t+1 can be expressed as follows:

$$([u_{t+1}{\tilde{r}}_{t+1}^{k}-\psi^{-(t+1)}a(u_{t+1})]P_{t+1}+(1-\delta )Q_{\bar{K}.t+1}){\bar{K}}_{t+1}\omega$$ (8)

which should be equivalent to the following equation:

$$(1+R_{t+1}^{k})Q_{\bar{k}.t}{\bar{K}}_{t+1}\omega$$ (9)

where $\left(1+R_{t+1}^{k}\right)$ is the average gross nominal rate of capital return for trust enterprise in period t+1. So we will have:

$$1+R_{t+1}^{k}=\frac{\left([u_{t+1}{\tilde{r}}_{t+1}^{k}-\psi^{-(t+1)}a(u_{t+1})]P_{t+1}+(1-\delta )Q_{\bar{k}.t+1}\right)}{Q_{K.t}}+\tau^{k}\sigma$$ (10)

In Eq 10, *τ*^*k*^ represents the fixed tax rate for capital. We assume that the trust enterprise can only finance a part of the capital balance by itself and therefore it will need financing from the financial market. The trust enterprise receives a loan from the bank for its external financing. According to the contract between the bank and the trust enterprise, based on the risk shock, *ω*, and based on the threshold amount of the risk shock, ${\bar{\omega }}_{t+1}$, the gross interest rate paid by the trust enterprise to the bank, which is denoted by *Z*_*t*+1_, is determined. The threshold level of risk shock is defined as follows:

$${\bar{\omega }}_{t+1}(1+R_{t+1}^{k})Q_{\bar{k^{\prime }}.t}K_{t+1}=Z_{t+1}B_{t+1},B_{t+1}=Q_{\bar{k^{\prime }}.t}{\bar{K}}_{t+1}-N_{t+1}$$ (11)

Which *B*_*t*+1_ shows the loans received by the trust enterprise from the bank. For trust enterprise which is $\omega \leq {\bar{\omega }}_{t+1}$, they cannot pay their loan to the bank. For the trust enterprise that goes bankrupt and cannot pay its loan, there will be the following conditions:

$$(1+R_{t+1}^{k})\omega Q_{\bar{k^{\prime }}.t}{\bar{K}}_{t+1}<B_{t+1}Z_{t+1}$$ (12)

In this situation, the bank must pay a fee for monitoring the trust enterprise, which will be equivalent to $\mu (1+R_{t+1}^{k})\omega Q_{\bar{k^{\prime }}.t}{\bar{K}}_{t+1}$, which in fact, the monitoring fee will be equivalent to a percentage of the gross income of the trust enterprise. Therefore, the cash value of trust enterprise assets will be equivalent to the following equation:

$$(1-\mu )(1+R_{t+1}^{k})\omega Q_{\bar{k^{\prime }}.t}{\bar{K}}_{t+1}$$ (13)

Now we assume that the trust enterprise exits the economy with the probability 1−*γ*_*t*+1_ and remains in the economy with the probability *γ*_*t*+1_ in the next period. Proportion Θ of the total wealth of the trust enterprise that exits the economy is spent on exiting the economy and the rest is paid to households. In each period, another number of trust enterprises enters the economy, so that the number of trust enterprises remains constant in all periods, and new trust enterprises bring net worth *W*^*e*^ into the economy. The average net worth of the trust enterprise will be as follows:

$${\bar{N}}_{t+1}=\gamma_{t}\{(1+R_{t}^{k})Q_{\bar{k^{\prime }}.t-1}{\bar{K}}_{t}-[1+R_{t}^{e}+\mu \frac{\int_{0}^{{\bar{\omega }}_{t}}\omega dF_{t}(\omega )(1+R_{t}^{k})Q_{\bar{k^{\prime }}.t-1}{\bar{K}}_{t}}{Q_{\bar{k^{\prime }}.t-1}{\bar{K}}_{t}-{\bar{N}}_{t}}](Q_{\bar{k^{\prime }}.t-1}{\bar{K}}_{t}-{\bar{N}}_{t})\}+W^{e})$$ (14)

In Eq 14, $Q_{\bar{k^{\prime }}.t-1}{\bar{K}}_{t}-{\bar{N}}_{t}=B_{t}$ is equivalent to the loan received in period t, and the values in the bracket of Eq 14 indicate the amount of loan paid by the trust enterprise to the bank. In fact, the trust enterprise’s payment amount to the bank, which is affected by the risk shock, will be as follows according to the amount of the loan received:

$$P_{t}^{e}=\mu \frac{\int_{0}^{{\bar{\omega }}_{t}}\omega dF_{t}(\omega )(1+R_{t}^{k})Q_{\bar{k^{\prime }}.t-1}{\bar{K}}_{t}}{Q_{\bar{k^{\prime }}.t-1}{\bar{K}}_{t}-{\bar{N}}_{t}}$$ (15)

The net worth of the trust enterprise shown at the end of period t+1 by Eq 14 is affected by two shocks that have different time structures. The financial wealth shock *γ*_*t*_ is realized at time t, but the risk shock caused by *σ*_*t*−1_ is realized at time t-1. In fact, b shows deviations in the efficiency of the trust enterprise. The risk shock will have a first-order vector autoregressive process as follows:

$$\sigma_{t}=\rho_{\sigma }\sigma_{t-1}+\lambda_{\sigma }^{oil}oil+\lambda_{\sigma }^{exch}exch$$ (16)

where $\lambda_{\sigma }^{oil}$ and $\lambda_{\sigma }^{exch}$ respectively indicate the role of oil price changes and exchange rate changes on the risk shock. The financial wealth shock also has a first-order autoregressive process.

### 3.3 Banks

We assume that banks are in a competitive market. The structure of the bank’s balance sheet at the end of period t can be seen in Table 1.

**Table 1:** Structure of the bank’s balance sheet at the end of the period.

Assets		Liabilities	
Reserves	*A*_*t*_	Household Deposits	${D_{t}^{h}=A}_{t}$
Working Capital Loans	$S_{t}^{\omega }$	Enterprises Deposits	$D_{t}^{f}=S_{t}^{\omega }$
Loans Paid to Trust Enterprises	*B*_*t*_	Short-term Securities	$D_{t}^{m}$
		Other Financial Securities	*T*_*t*−1_

In the following, in order to better express the bank sector in the model, we will explain the bank sector in the two parts of loans and deposits, and in the following, we will first explain the part of loans.

The bank pays two types of loans. One type of those loans are working capital loans to enterprises that produce intermediate goods, which we denote by $S_{t}^{\omega }$. This type of loan is paid at the beginning of the period and its maturity is until the end of the same period. If we consider the interest rate of this loan as *R*_*t*_, then the bank’s interest income from granting this type of loan is equal to:

$$\left(1+R_{t})S_{t}^{\omega }=(1+R_{t})(\varphi_{l}W_{t}l_{t}+\varphi_{k}P_{t}{\tilde{r}}_{t}^{k}K_{t}\right)$$ (17)

The second type of loan is the loans paid to the trust enterprise. This type of loan is paid at the end of the period and its maturity will be in future periods. Therefore, the loan payment at the end of period t will be *B*_*t*+1_. The average unconditional interest rate (regardless of the risk conditions) of this type of loan is denoted by $R_{t+1}^{e}$, and the gross interest rate paid by the trust enterprise will be *Z*_*t*+1_. The contract between the trust enterprise and the bank has two parameters of the loan payment and the second is the interest rate without loan default, or *Z*_*t*+1_ (which will be equivalent to ${\bar{\omega }}_{t+1}$). These two parameters are obtained by maximizing the net worth of the trust enterprise, taking into account the condition of zero profit for the bank, which will be as follows:

$$MaxE_{t}\{[1-\Gamma_{t}({\bar{\omega }}_{t+1})]\frac{1+R_{t+1}^{k}}{1+R_{t+1}^{e}}(B_{t+1}+N_{t+1})+\eta_{t+1}([\Gamma_{t}({\bar{\omega }}_{t+1})-\mu G_{t}({\bar{\omega }}_{t+1})]\frac{1+R_{t+1}^{k}}{1+R_{t+1}^{e}}(B_{t+1}+N_{t+1})-B_{t+1})\}$$ (18)

where *η*_*t*+1_ represents the Lagrange coefficient.

In order to cover their credit risk, banks consider the loan interest rate higher than the risk-free interest rate, which is the same as the deposit absorption rate from the household. Also, like the banking system in Iran, we assume that the interest rate paid to depositors (households) is not affected by the risk shock. The total amount of loans at the end of period t, which we denote by $B_{t}^{Tot}$, is the sum of working capital loans and trust enterprise loans, which will be as follows:

$$B_{t}^{Tot}=\varphi_{l}W_{t}l_{t}+\varphi_{k}P_{t}{\tilde{r}}_{t}^{k}K_{t}+B_{t+1}$$ (19)

As a financial intermediary between the households and the production sector, the bank creates three types of liability namely deposits, short-term tradable securities and other securities. The bank uses deposits and short-term securities for liquidity and lending services. Similar to Chari et al. [69]’s model, we assume that the bank uses a technology function to convert labor force $l_{t}^{b}$, capital $K_{t}^{b}$, and excess reserves $E_{t}^{r}$, in order to provide liquidity provision services for trust enterprises and enterprises producing intermediate goods. which is as follows:

$$\frac{D_{t}^{h}+D_{t}^{f}+\varsigma D_{t}^{m}}{P_{t}}=x_{t}^{b}{\left({\left(K_{t}^{b}\right)}^{\alpha }{\left(z_{t}l_{t}^{b}\right)}^{1-\alpha }\right)}^{\zeta }{\left(\frac{E_{t}^{r}}{P_{t}}\right)}^{1-\zeta }$$ (20)

that $D_{t}^{h}$ and $D_{t}^{f}$ represent household and enterprise deposits in the bank, and $D_{t}^{m}$ also represents short-term tradable bonds, and *ς* is also a positive value, as well as 0<*α*<1. $x_{t}^{b}$ and *ζ*∈(0.1) are random processes so that $x_{t}^{b}$ represents the deposit technology shock and *ζ* is the shock that the central bank uses to demand more reserves from banks. $x_{t}^{b}$ will have a first-order vector autoregressive process as follows:

$$\varepsilon_{t}^{x^{b}}≅iid\;x_{t}^{b}=\rho_{x^{b}}x_{t-1}^{b}+\varepsilon_{t}^{x^{b}}$$ (21)

In this model, monetary policy is applied through the credit channel (lending channel) through shock $x_{t}^{b}$. Now we assume that household deposits are equal to the total cash reserves of the bank, so that:

$${D_{t}^{h}=A}_{t}$$ (22)

and enterprises’ deposit is spent on working capital loans, so that:

$$D_{t}^{f}=S_{t}^{\omega }$$ (23)

Banks are required to maintain minimum legal reserves, which we show as the ratio of legal reserves, that is *τ*. Therefore, the total cash reserves (*A*_*t*_) after deducting the minimum reserves can be used for the liquidity required by enterprises:

$$E_{t}^{r}=A_{t}-\tau (D_{t}^{f}+D_{t}^{h})$$ (24)

The interest rate of deposits is considered as $R_{t}^{a}$, so that the bank’s interest expenses are financed through the interest income that results from the loans and it can be said that the interest rate that producers of intermediate goods pay for working capital loans is higher than the interest rate received for deposits, so the interest rate of working capital loans will be equal to $R_{t}^{a}+R_{t}$ and therefore the net interest rate paid by enterprises for working capital loans will be equal to *R*_*t*_. Short-term tradable bonds $D_{t}^{m}$, and other financial assets *T*_*t*_, which are issued at the end of the production period, are used to finance the loans of trust enterprises, so that:

$$D_{t+1}^{m}+T_{t}=B_{t+1}$$ (25)

Tradable bonds and other financial assets will be different from each other in terms of the interest rates paid to them because the interest rate paid to the household will be for tradable bonds $R_{t+1}^{m}$ and the interest rate paid to other financial assets $R_{t+1}^{T}$. The noteworthy point here is that $R_{t+1}^{m}$ and $R_{t+1}^{T}$ are conditional on the shocks up to time t, but they will be unconditional to the shocks of period t+1. The profit function of the bank can be expressed as the following equation:

$$\Pi_{t}^{b}=(1+R_{t}+R_{t}^{a})S_{t}^{\omega }+(1+R_{t}^{e})B_{t}+A_{t}+T_{t}+D_{t+1}^{m}-B_{t+1}-(1+R_{t}^{a})(D_{t}^{f}+D_{t}^{h})-(1+R_{t}^{m})D_{t}^{m}-(1+R_{t}^{T})T_{t-1}-[(1+{\varphi_{k}R}_{t})P_{t}{\tilde{r}}_{t}^{k}K_{t}^{b}]-[(1+{\varphi_{k}R}_{t})W_{t}l_{t}^{b}]$$ (26)

### 3.4 Households

In our model, households are consumers of final goods, savers and suppliers of labor to enterprises and banks. Labor wage adjustment is done based on Calvo model [2]. The preferences of the jth household can be shown as follows:

$$E_{t}^{j}\sum_{l=0}^{\infty }\beta^{l}(u(C_{t+l}-bC_{t+l-1})-\varphi_{l}\frac{h_{j.t+l}^{1+\sigma_{L}}}{1+\sigma_{L}}-H\left(\frac{M_{t+l}P_{t+l-1}}{M_{t+l-1}P_{t+l}}\right)-\upsilon \frac{{\left({\left(\frac{(1+\tau^{c})P_{t+l}C_{t+l}}{M_{t+l}}\right)}^{(1-\chi_{t+1})\theta }{\left(\frac{(1+\tau^{c})P_{t+l}C_{t+l}}{D_{t+l}^{h}}\right)}^{\left(1-\chi_{t+1})(1-\theta \right)}{\left(\frac{(1+\tau^{c})P_{t+l}C_{t+l}}{D_{t+1}^{m}b}\right)}^{\chi_{t+1}}\right)}^{1-\sigma_{q}}}{1-\sigma_{q}})$$ (27)

Where $E_{t}^{j}$ represents the conditional expectations of household j based on information up to time t. Parameters *C*_*t*_, *h*_*j*.*t*_, *τ*^*c*^, *ζ*_*c*.*t*_, *M*_*t*_ and $D_{t}^{h}$ respectively represent consumption, working hours at time t, consumption tax, exogenous shock of preferences at time t, household money and household deposit in the bank. In Eq 27, the function H shows the real cost of keeping money, and the function H will be minimum in conditions where the growth rate of the money volume is equivalent to the long-term sustainable growth rate. Household liquidity preferences are shown by two parameters *υ* and *θ*, and *χ*_*t*_ also represents the shock related to other types of liquidity maintenance (short-term tradable bonds). By using *b*>0 in Eq 27, the preferences of household consumption habits are also taken into account. The household starts period t by holding money $M_{t}^{b}$. The household will face the following constraint to split between cash *M*_*t*_ and bank deposits *A*_*t*_:

$$M_{t}^{b}-(M_{t}+A_{t})\geq 0$$ (28)

We denote the money injection during period t by *X*_*t*_, so that the household will have cash equivalent to *M*_*t*_+*X*_*t*_ at the end of period t. In this case, banking services will only be a function of *M*_*t*_ because *X*_*t*_ will be realized at the end of period t and we also assume that households receive labor wages and interest income from the interest rate of bank deposits at the end of period t and therefore cannot use them during period t to purchase goods. Sources of household income and household expenses in period t and their related equations are shown in Table 2.

**Table 2:** Sources of household income and expenditure in Period t.

Income	
**Description**	**Equation**
Income from Labor Wages	(1−*τ*^*l*^)*W*_*j*.*t*_*h*_*j*.*t*_
Cash with Household Plus Cash Injection During Period t	*M*_*t*_+*X*_*t*_
Interest Income from Bank Deposits	$(1+R_{t}^{a})D_{t}^{h}$
Interest Rate and Principal of Tradable Securities and Other Financial Assets	$(1+R_{t}^{m})D_{t}^{m}$ and $(1+R_{t}^{T})D_{t}^{T}$
Cash Reserves of Banks	*A*_*j*.*t*_
Lump Sum Payments by Government to Household	*Lump*_*t*_
Net worth of Trust Enterprises that Exit the Economy at the End of Period t	$(1-\Theta )(1-\gamma_{t})({\bar{N}}_{t+1}-W^{e})/\gamma_{t}$
Profit from Enterprises Producing Final and Capital Goods, Intermediate Goods and Banks	-
**Expense**	
Payment for Consumer Goods	(1+*τ*^*C*^)*P*_*t*_*C*_*t*_
Cash Balance Required for Period t+1	$M_{t+1}^{b}$
Buying Short-term Tradable Bonds	$D_{t+1}^{m}$
Buying Other Financial Assets	*T*_*t*_
Payment to New Trust Enterprise	*W* ^*e*^

Therefore, according to the sources of income and payments of the household, the following equation can be defined:

$$(1+R_{t}^{a})(M_{t}^{b}-M_{t})+X_{t}-T_{t}-D_{t+1}^{m}-(1+\tau^{C})P_{t}C_{t}+(1-\Theta )(1-\gamma_{t})({\bar{N}}_{t+1}-W^{e})/\gamma_{t}-W^{e}+{Lump}_{t}+(1+R_{t}^{T})T_{t-1}+(1+R_{t}^{m})D_{t}^{m}+(1-\tau^{l})W_{j.t}h_{j.t}+M_{t}+X_{t}+\Pi_{t}+A_{j.t}\geq M_{t+1}^{b}\geq 0$$ (29)

### 3.5 Final goods market settlement conditions

The final goods market settlement conditions will be as follows:

$$\mu \int_{0}^{{\bar{\omega }}_{t}}\omega dF(\omega )(1+R_{t}^{k})\frac{Q_{\bar{k^{\prime }}.t-1}{\bar{K}}_{t}}{P_{t}}+\frac{a(u_{t})}{\psi^{t}}{\bar{K}}_{t}+\frac{\Theta (1-\gamma_{t})V_{t}}{P_{t}}+G_{t}+C_{t}+(\frac{1}{\left(\psi^{t}\mu_{\psi .t}\right)})I_{t}\leq Y_{t}+Y_{t}^{0}$$ (30)

The first term in Eq 30 represents a part of the final production that is used to monitor banks. The second term of Eq 30 shows the cost of capital consumption. The third term of Eq 30 includes 1−*γ*_*t*_ percentage of trust enterprises that leave the economy in period t. The fourth term of this equation also represents government consumption, which will be in the form of Eq 31:

$$G_{t}=z_{t}^{*}g_{t}$$ (31)

where *g*_*t*_ has a random process, so in this case, the model has a balanced growth rate. Also, *C*_*t*_ represents the total household consumption of the final good. The last term on the left side of Eq 30 shows the amount of final goods used in the production of investment goods *I*_*t*_. On the right side of Eq 30, $Y_{t}^{0}$ is the amount of oil revenue. In the following, in order to establish the relationship between total production parameters and total investment and total labor supplied by the household, we use the following equation:

$$Y_{t}+Y_{t}^{0}=G_{t}+C_{t}+(\frac{1}{\left(\psi^{t}\mu_{\psi .t}\right)})I_{t}$$ (32)

### 3.6 Monetary authority

Another part of the economy is the government and the central bank. To model this section, we use the structure of the model provided by Tavakolian and Komijani [68]. We define the reaction function of the monetary authority as follows:

$$x_{t}=\rho_{x}x_{t-1}+\lambda^{\pi }(\pi_{t}-\pi_{t}^{target})+\lambda^{y}(y_{t}-y_{t}^{*})+\varepsilon_{t}^{x},\pi_{t}^{target}=\rho_{\pi }\pi_{t-1}^{target}+\varepsilon_{t}^{target}$$ (33)

where $\varepsilon_{t}^{target}$ is the shock to the monetary policymaker’s target inflation. The monetary shock that we showed with $\varepsilon_{t}^{x}$ in Eq 33 will also have a first-order vector autoregressive process as follows:

$$\varepsilon_{t}^{x}=\rho_{x}^{*}\varepsilon_{t-1}^{x}+\varepsilon_{t}^{mb}$$ (34)

It should be noted that this reaction function of the monetary policy maker will be a kind of policy rule. As we know, the objective function based on the bank interest rate is mainly used to check the Monetary Authority section. A very important point that should be mentioned in this section is that in Iran’s economy, the bank interest rate is fixed and is determined by the central bank for a certain period of time, and this makes us not use the bank interest rate to determine the objective function.

## 4. Results

In this section, the proposed model for the case study is evaluated. Based on this, a real dataset related to Iran’s macroeconomics has been extracted.

After extracting the first-order conditions in the maximization of the objective functions in the households, enterprises (enterprises producing intermediate goods and enterprises producing final and capital goods), trust enterprises, banks and monetary authority, the equations are Linear-logarithmically adjusted. Part of the model’s calibrated parameters, which are compiled based on the findings of other studies, are given in Table 3, and other model parameters are obtained through solving the model in terms of other parameters.

**Table 3:** Calibrated parameters of the model.

Parameter	Description	Source	Based on Model
**Household Sector Parameters**			
*β*	Time Value Discount Rate	Tavakolian and Kamijani [68]	0.96
*σ*_*L*_	Disutility Sensitivity of Work	Tavakolian and Kamijani [68]	2.2156
*υ*	Utility Weight of Money	Tavakolian and Kamijani [68]	0.002
*σ*_*q*_	Utility Sensitivity of Money	Tavakolian and Kamijani [68]	2.24
*θ*	Power of Money in Utility Function	Tavakolian and Kamijani [68]	2.24
*χ*	power of deposits in Utility Function	Christiano et al. [65]	0.4
*b*	Consumption Habit Preferences	Shahhosseini and Bahrami [70]	0.63
*λ*_*ω*_	Labor Supply	Shahhosseini and Bahrami [70]	1.05
**Producers Sector (Final and Intermediate Goods)**			
*μ*_*z*_	Economic Growth Rate	Shahhosseini and Bahrami [70]	1.028
*φ*_*l*_	Part of Wage That Is Financed	Christiano et al. [65]	0.75
*φ*_*k*_	Part of Capital Lease That Is Financed	Christiano et al. [65]	0.75
*δ*	Capital Depreciation Rate	Shahhosseini and Bahrami [70]	0.023
*α*	Power of Capital in Production Function	Shahhosseini and Bahrami [70]	0.675
*λ*_*f*_	Technology Shock in Intermediate Goods	Tavakolian and Kamijani [68]	1.2
*Φ*	Fixed Cost in Intermediate Goods	Tavakolian and Kamijani [68]	0.07
**Trust Enterprises Sector**			
*γ*	Percentage of Trust Enterprises that Remain in Economy	Christiano et al. [65]	0.976
*μ*	Percentage of Profit of Trust Enterprises, Which Is Cost of Monitoring.	Christiano et al. [65]	0.94
$F(\bar{\omega })$	Percentage of Businesses that Go Bankrupt in A Period	Christiano et al. [65]	0.0576
*Var*(log(*ω*))	Logarithm Variance of Productivity Parameter	Christiano et al. [65]	0.009
**Banks Sector**			
*ζ*	Excess Reserves of Bank Deposits	Christiano et al. [65]	0.96
*x* ^*b*^	Deposit Technology Shock	Christiano et al. [65]	90.5
**Monetary Authority Sector**			
*x*	Growth Rate of Money Supply	Tavakolian and Kamijani [68]	0.009
*ρ*_*x*_	Autoregressive Coefficient of Monetary Growth	Tavakolian and Kamijani [68]	0.798
*ρ*_*π*_	Autoregressive Coefficient of Target Inflation	Tavakolian and Kamijani [68]	0.8912
*λ* ^*π*^	Coefficient of Importance of Inflation in Monetary Reaction Function	Tavakolian and Kamijani [68]	-1.067
*λ* ^*y*^	Coefficient of Importance of Production in Monetary Reaction Function	Tavakolian and Kamijani [68]	-2.4999
*σ*_*m*_	Standard Deviation of Monetary Policy Shock	Tavakolian and Kamijani [68]	0.0109

Here we should briefly refer to the data used in Table 4, which is shown in the form of four variables of Output, Consumption, Investment and Inflation. It is very important to mention that the data related to these four variables are available on the websites of the Central Bank of Iran and the World Bank and respectively, it was extracted from the variables of gross national product in terms of economic activities, consumption expenses of the private and public sector, net capital stock and inflation in the Iranian economy during the years 1986 to 2021.

**Table 4:** Summary of data used in the presented model.

Year	Output	Consumption	Investment	Inflation
1986	$209.24B	$0.32B	$6.08B	0.237
1987	$133.94B	$0.84B	$6.91B	0.277
1988	$122.97B	$1.23B	$7.50B	0.289
1989	$120.42B	$2.14B	$7.89B	0.174
1990	$124.76B	$3.87B	$9.18B	0.09
1991	$91.63B	$5.27B	$10.91B	0.207
1992	$80.51B	$8.91B	$13.41B	0.244
1993	$63.76B	$10.17B	$17.41B	0.229
1994	$71.51B	$14.41B	$19.51B	0.352
1995	$95.92B	$19.99B	$23.11B	0.494
1996	$119.85B	$25.62B	$24.15B	0.232
1997	$113.67B	$33.50B	$29.39B	0.173
1998	$110.19B	$37.19B	$34.41B	0.181
1999	$114.12B	$41.29B	$35.43B	0.201
2000	$109.78B	$45.13B	$41.85B	0.126
2001	$127.17B	$47.77B	$45.91B	0.114
2002	$128.98B	$50.39B	$46.17B	0.158
2003	$153.83B	$51.34B	$51.19B	0.156
2004	$189.73B	$54.70B	$54.13B	0.152
2005	$226.04B	$55.41B	$57.13B	0.104
2006	$266.41B	$61.09B	$59.61B	0.119
2007	$350.83B	$66.97B	$63.90B	0.184
2008	$413.69B	$67.07B	$71.13B	0.254
2009	$416.27B	$67.13B	$74.59B	0.108
2010	$486.88B	$68.93B	$76.61B	0.124
2011	$626.59B	$70.15B	$79.43B	0.215
2012	$648.24B	$71.17B	$80.62B	0.305
2013	$495.91B	$73.18B	$83.21B	0.347
2014	$462.75B	$73.96B	$85.29B	0.156
2015	$408.51B	$75.31B	$89.14B	0.119
2016	$458.48B	$76.87B	$92.17B	0.09
2017	$487.22B	$79.46B	$96.87B	0.096
2018	$333.27B	$80.17B	$107.15B	0.312
2019	$284.42B	$81.39B	$109.37B	0.412
2020	$240.40B	$83.55B	$116.93B	0.417
2021	$360.91B	$85.14B	$117.28B	0.462

Tests and post-testing of the results obtained from the model should be reported with the actual observation values as shown in Table 5.

**Table 5:** Tests and post-testing of the results.

Variable Name	Output	Consumption	Investment	Inflation
Standard Deviation of the Real Variable	0.05	0.03	0.17	0.01
Standard Deviation of the Simulated Variable	0.08	0.07	0.01	0.04
Autocorrelation Coefficient of the Real Variable with Lag 1	0.73	0.9	0.67	0.28
Autocorrelation Coefficient of Simulated Variable with Lag 1	0.82	0.95	0.98	0.91
Autocorrelation Coefficient of the Real Variable with Lag 2	0.84	0.82	0.43	0.15
Autocorrelation Coefficient of Simulated Variable with Lag 2	0.65	0.87	0.93	0.73

**Fig 3:**
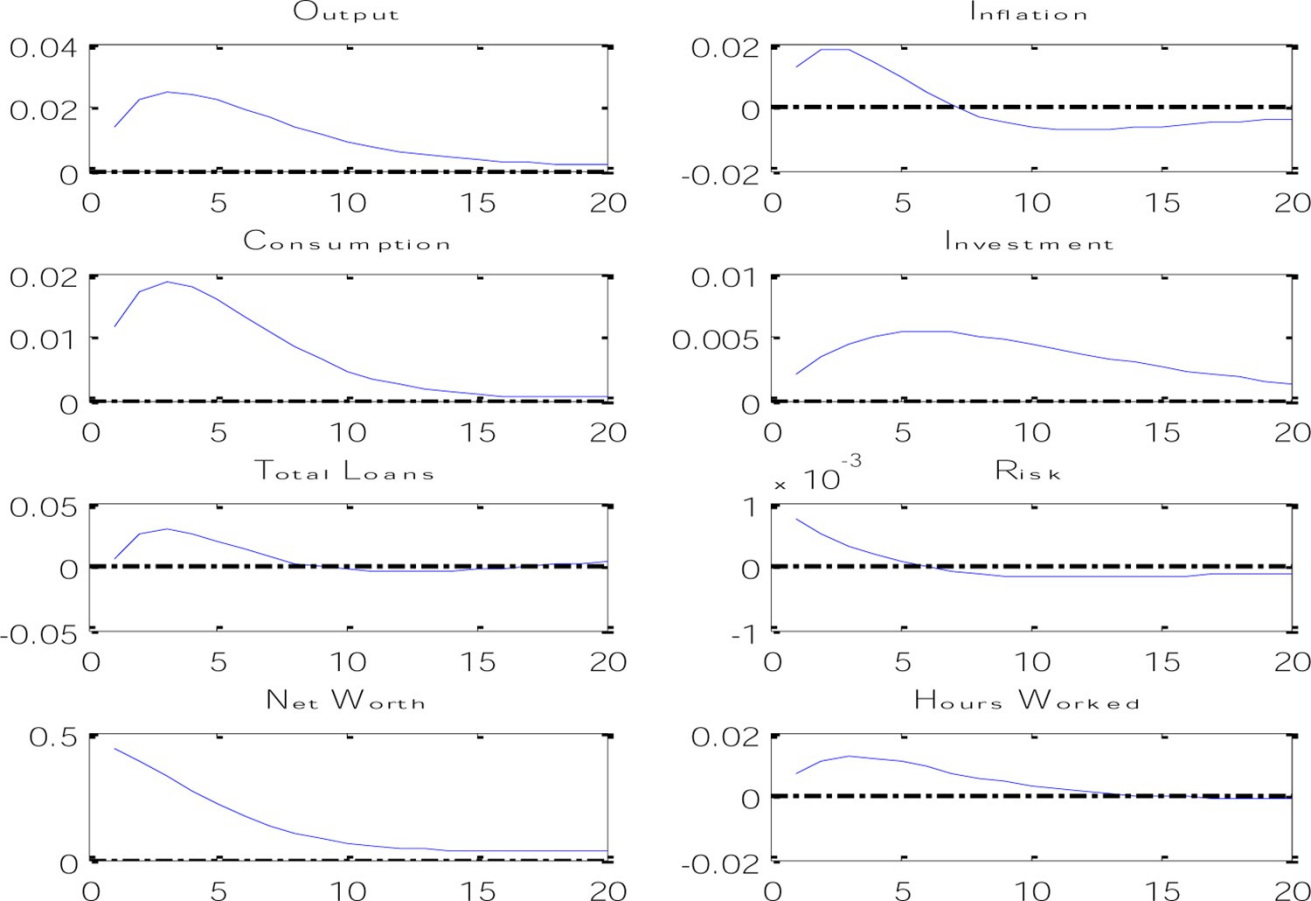
Effects of monetary policy shock on economic variables.

In DSGE models, unlike macrometric models, model parameters are calibrated and introduced to the model. Then, according to the parameters as well as the information provided through the equations, the model simulates the variables through processes and presents some characteristics of the simulated variables such as standard deviation and correlation coefficients. The comparison between the characteristics of the filtered variables of the real world (based on seasonal data) and the simulated variables can be a measure of the success of the designed model, and the results of the model showed that the designed DSGE model is suitable for Iran’s economy. After calibrating the model and tests, we will examine the test of the main hypotheses of the research. The hypothesis of this article is to investigate the existence of the credit channel and the risk channel of expansionary monetary policy using DSGE models, which in Fig 3 shows the effects of an expansionary monetary policy on economic variables.

According to the results obtained from the instantaneous reaction functions of macroeconomic variables against an expansionary monetary policy shock through an increase in the amount of money, it can be said that an increase in the amount of money in one period causes the cash balance of the household to increase in the next period and as a result, it causes an increase in the volume of household deposits as well as an increase in household consumption. An increase in deposits causes an increase in the lending power of banks, and as a result, an increase in the investment volume of trust enterprises. With the increase in investment, the amount of capital in the economy has increased, which causes an increase in the net worth of trust enterprises. On the other hand, the increase in capital level makes trust enterprises need more bank loans and since the relative share of trust enterprises loans increases compared to total bank loans, it causes the risk of loans in the banking system to increase because other loans do not have risks except working capital loans. On the other hand, we know that an expansionary monetary policy causes an increase in banking risk, so Fig 3 shows that by increasing the amount of money, it has caused a decrease in the cost of financing trust enterprises and also an increase in the capital return rate, which causes that Banks give more loans to projects with higher returns, and since higher returns bring more risk, the risk of the banking system increases. On the other hand, with the increase in consumption and investment in the economy, the amount of production will increase, and this means an increase in the number of working hours. Since the increase in production was due to the increase in demand in the economy, this also causes an increase in inflation.

It is worth mentioning that the Monetary Transmission Mechanism on the side of the credit channel does not only affect the demand for loans, but it is able to affect the supply of bank credits in the market and consequently affect investment and consumption as well. In other words, Monetary Transmission Mechanism on the side of the credit channel will be effective on both borrowers and lenders. Due to the specific role of banks, some borrowers will not have access to credit markets unless they borrow from banks. If there is no perfect substitution between bank micro-deposits and other sources of funding, expansionary monetary policy increases lending power such as bank reserves and bank deposits. The role of banks as lenders to classes of bank borrowers causes an increase in loans that will increase investment expenses. Therefore, it can be said that by applying an expansionary monetary policy shock, the volume of production, inflation, private sector consumption, investment, net worth in the economy and the amount of working hours will increase, as well as the total amount of loans and the risk of lending. The obtained results show that in addition to an expansionary monetary policy shock in Iran’s economy through channels such as interest rate, wealth-based channel, and consumption-based channel, it has an effect on real production and price level, this shock will have an effect on macroeconomic variables through the credit channel by increasing loans and the risk channel.

**Fig 4:**
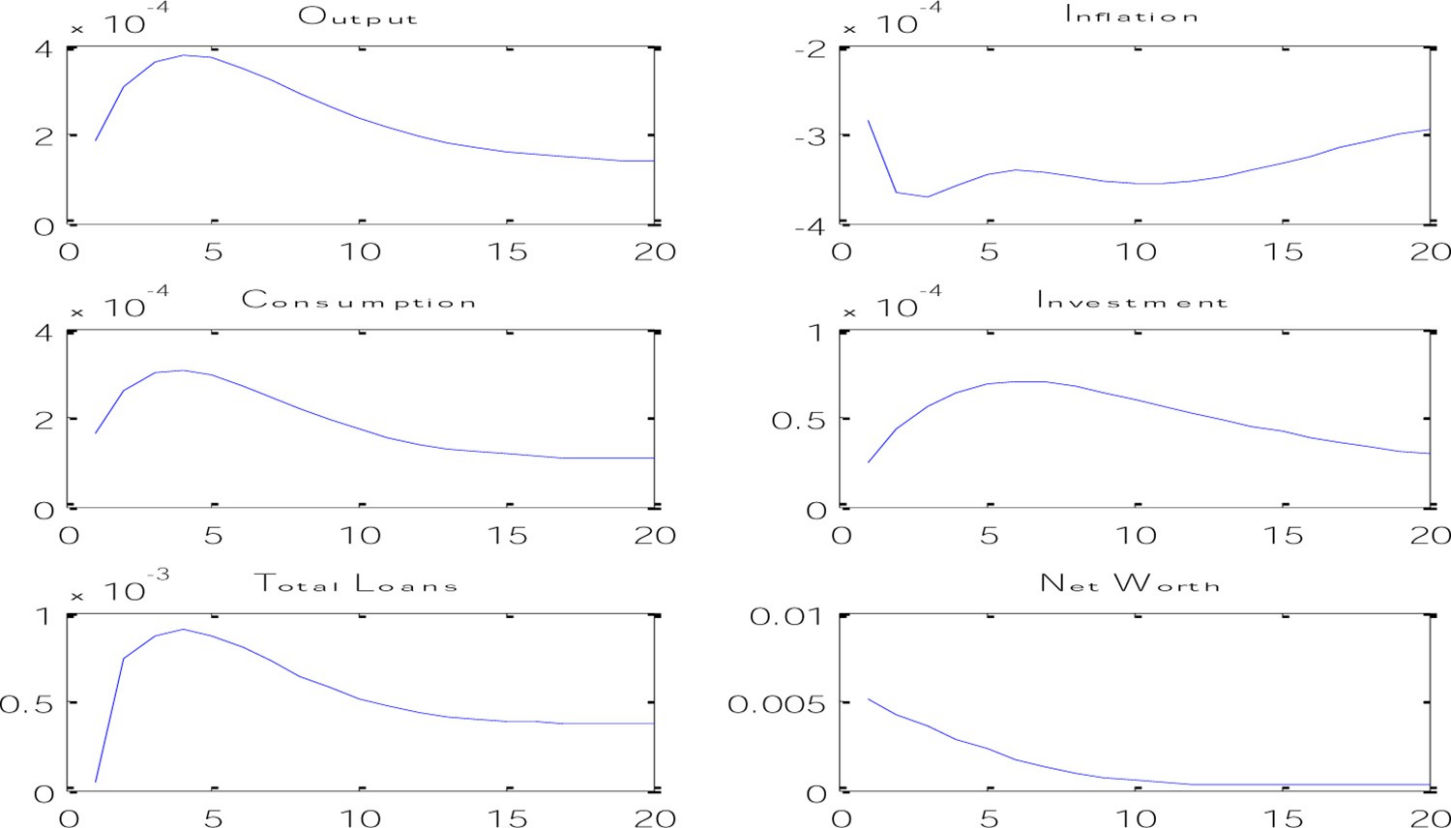
Credit shock effects on economic variables.

According to Fig 4, it can be said that by applying a credit shock through an increase in lending power, banks will pay more loans with the same amount of deposits they had before, which causes an increase in the total volume of loans. and as a result causes an increase in the volume of investment and therefore an increase in production and consumption in the economy. An increase in investment causes an increase in the capital level of the next period, which causes an increase in the net worth of trust enterprises. On the other hand, because the increase in production has happened through the increase in lending power of banks, and in fact, the increase in the productivity of the banking system has happened, this issue will cause a decrease in inflation. This type of credit shock can be expressed in the increase of lending power of banks through the sale of surplus fixed assets of banks or the collection of outstanding debts of banks in the Iranian economy, which will reduce inflation and increase production in the economy.

**Fig 5:**
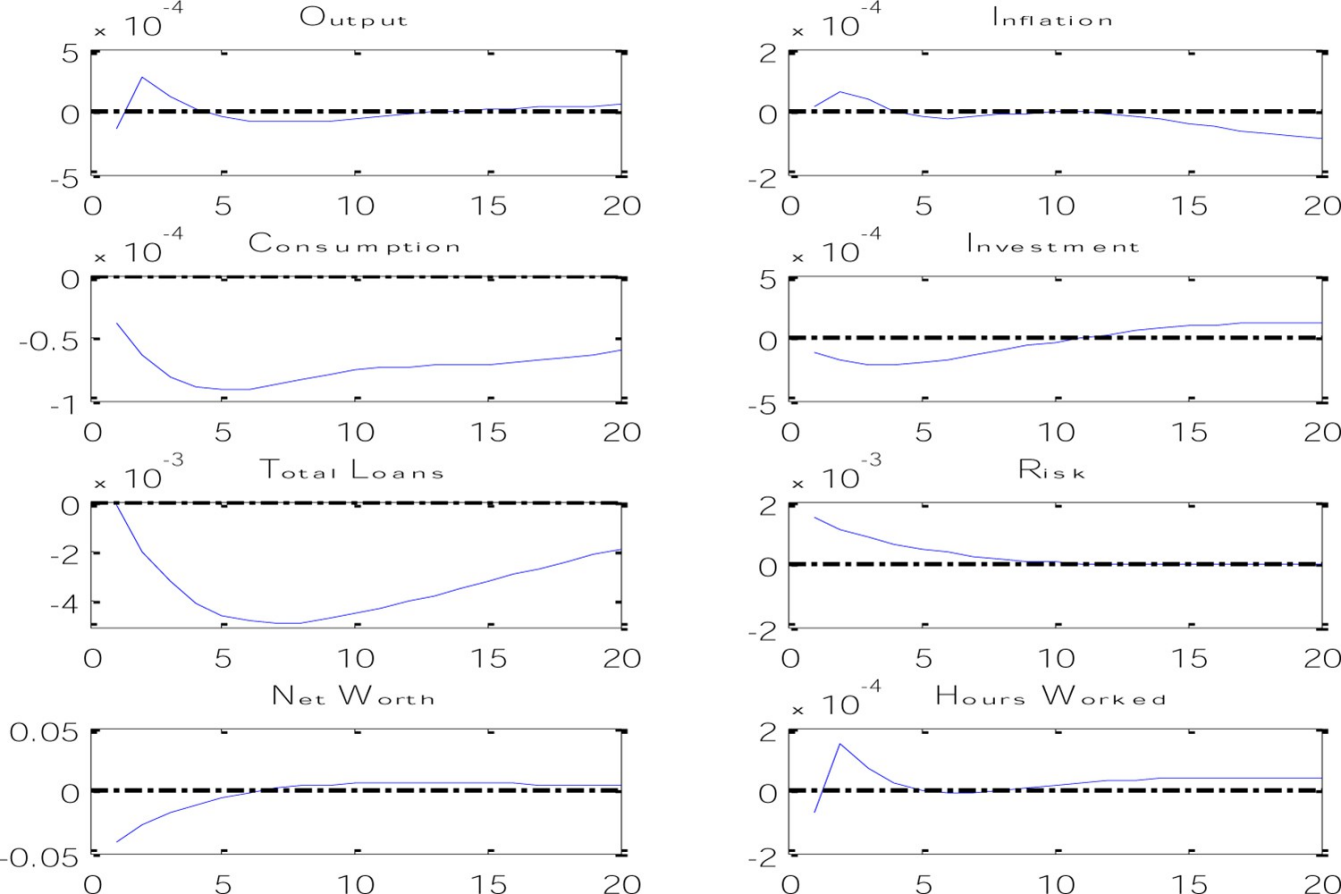
Effects of risk shock on economic variables.

In Fig 5, the effects of the risk shock on the variables of the model are shown. According to the results obtained from the instantaneous response functions in Fig 4, it can be said that with the increase in risk in the banking system, the net worth will decrease at first, because with the increase in risk, the amount of monitoring of trust enterprises projects has increased and this issue will cause a decrease in the income of trust enterprises, which in turn will reduce the demand for bank loans, and such a banking system will also limit its loan supply in risky conditions.

Less net worth means that in reality, banks have received less collateral to pay loans, so the amount of losses caused by adverse selection is more for these banks. The decrease in the net amount of wealth has increased the probability of adverse selection and will lead to a decrease in lending and investment expenses. A decrease in wealth means that business owners have less assets to pledge, so they have higher incentives to choose risky projects. This issue increases the possibility of banks not being able to recover loans, so the decrease in the amount of net worth of enterprises will lead to a decrease in lending and a decrease in investment expenses. By reducing the net worth of trust enterprises and reducing bank loans, the amount of investment in the economy will decrease, which will cause the production level of the economy to increase at first but then decrease.

As shown in Fig 5, with the increase in banking risk, inflation increased at first, but after several periods it decreased, which can be attributed to the decrease in private sector consumption. If the level of consumption increases due to the increase in the risk of the banking system that happened in the Iranian economy in recent years, the pressure on the demand side of the economy will increase and the inflation rate will increase. In fact, it should be said that the increase in the risk of the banking system in recent years has caused more pressure on the increase in the inflation rate.

**Fig 6:**
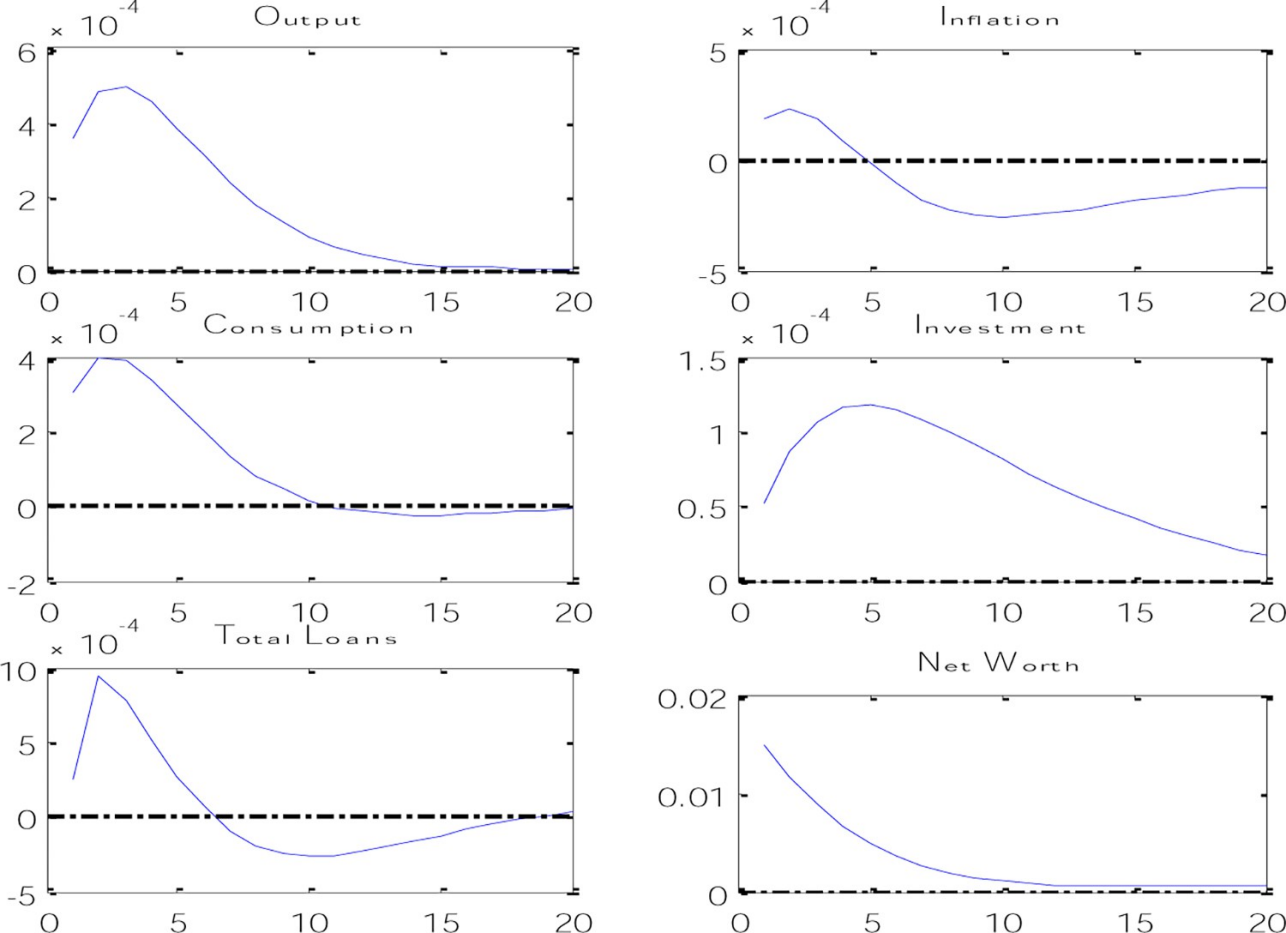
Effects of consumer liquidity preferences on economic variables.

Now we want to show the effects of consumer liquidity preferences on the variables of Iran’s economy in Fig 6.

**Fig 7:**
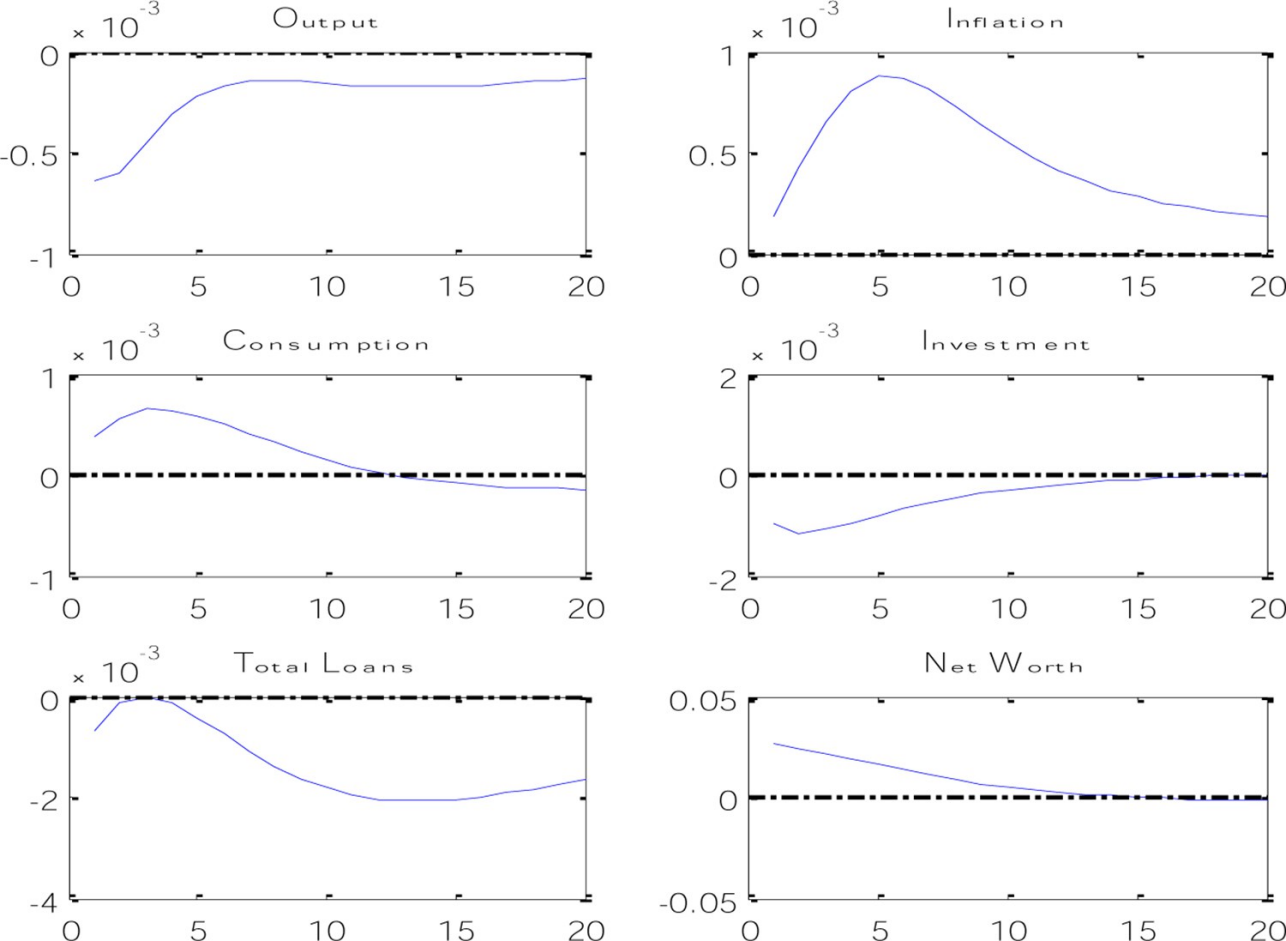
Effects of negative technology shock on economic variables.

The shock effects of household liquidity preferences on economic variables show that this shock causes an increase in the lending power of the banking system, which causes an increase in investment in the economy as well as an increase in net worth. The increase in household demand for non-cash assets, which manifests itself in the form of an increase in investment, will cause an increase in production and consumption level, as well as a decrease in inflation. Also, the effects of the negative shock of technology (increasing the cost of equipment installation) on the variables of Iran’s economy are shown in Fig 7.

According to the instantaneous reaction functions for the shock of increasing the cost of equipment installation, the level of investment decreases, which causes a decrease in production. A decrease in investment causes a decrease in loan demand. Due to the decrease in the supply of bank loans, the risk of the banking system has decreased, which increases net worth, which increases the level of private sector consumption.

Finally, in Table 6, the numbers obtained from the proposed model about the 8 investigated parameters are shown under Monetary Policy, Credit, Risk, Consumer Liquidity Preferences and Negative Technology shocks respectively.

**Table 6:** Summary of obtained results (related to the Figs 3–7).

**Monetary Policy Shock**					
		**5**	**10**	**15**	**20**
**Output**		0.0245	0.0093	0.0033	0.0017
**Inflation**		0.0087	-0.0064	-0.0049	-0.0028
**Consumption**		0.0123	0.0048	0.0017	0.0003
**Investment**		0.0053	0.0042	0.0031	0.0018
**Total Loans**		0.0223	-0.0012	-0.0006	-0.0002
**Risk**		0.112e-3	-0.0113e-3	-0.0132e-3	-0.0181e-3
**Net Worth**		0.2314	0.1137	0.0747	0.0697
**Hours Worked**		0.0113	0.0048	0.0009	0.0003
**Credit Shock**					
		5	10	15	20
**Output**		3.4141e-4	1.9314e-4	1.7137e-4	1.5690e-4
**Inflation**		-3.6142e-4	-3.7481e-4	-3.3947e-4	-2.9145e-4
**Consumption**		2.2617e-4	1.8751e-4	1.4450e-4	1.2793e-4
**Investment**		0.7139e-4	0.5222e-4	0.3870e-4	0.3384e-4
**Total Loans**		0.8735e-3	0.5449e-3	0.4238e-3	0.4008e-3
**Net Worth**		0.0023	0.0093	0.0071	0.0068
**Risk Shock**					
		**5**	**10**	**15**	**20**
**Output**		-0.7158e-4	-0.6481e-4	0.0316e-4	0.4147e-4
**Inflation**		-0.2143e-4	-0.0019e-4	-0.5138e-4	-0.9277e-4
**Consumption**		-0.8937e-4	-0.7813e-4	-0.6781e-4	-0.5469e-4
**Investment**		-2.2316e-4	-0.0731e-4	0.6138e-4	1.2350e-4
**Total Loans**		-5.3152e-3	-5.1786e-3	-3.4100e-3	-2.0011e-3
**Risk**		0.5113e-3	0.0015e-3	0.0003e-3	0.0001e-3
**Net Worth**		-0.0011	0.0029	0.0061	0.0033
**Hours Worked**		-0.0138e-4	0.0337e-4	0.3111e-4	0.3349e-4
**Consumer Liquidity Preferences Shock**					
		**5**	**10**	**15**	**20**
**Output**		3.2511e-4	0.7135e-4	0.0019e-4	0.0003e-4
**Inflation**		-0.0023e-4	-2.7111e-4	-1.6922e-4	-0.9158e-4
**Consumption**		2.6111e-4	0.0115e-4	-0.3116e-4	-0.0231e-4
**Investment**		1.1237e-4	0.8446e-4	0.3924e-4	0.2151e-4
**Total Loans**		3.6612e-4	-2.9100e-4	-1.2013e-4	0.0093e-4
**Net Worth**		0.0048	0.0013	0.0008	0.0007
**Negative Technology Shock**					
		**5**	**10**	**15**	**20**
**Output**		-0.1818e-3	0.1702e-3	-0.2366e-3	-0.1139e-3
**Inflation**		0.9136e-3	0.4457e-3	0.2843e-3	0.2183e-3
**Consumption**		0.6194e-3	0.2012e-3	-0.1431e-3	-0.2462e-3
**Investment**		-0.8450e-3	-0.3567e-3	-0.0635e-3	-0.0119e-3
**Total Loans**		-0.2431e-3	-1.8151e-3	-2.1882e-3	-1.7449e-3
**Net Worth**		0.0135	0.0031	0.0015	0.0007

## 5. Conclusions and future research directions

Monetary policy and determining the effect of monetary measures on macroeconomic variables such as economic growth rate, inflation rate, and employment are among the topics that have been explored among economists for decades. There has been extensive literature about the effect of monetary changes on economic target variables such as inflation and production, which is generally referred to as Monetary Transmission Mechanism. Monetary transmission channels can be divided into traditional channels of monetary effects transmission and credit channels and monetary policy risk. According to the empirical studies that have been conducted, it can be said that an expansionary monetary policy causes an increase in banks’ risk, and on the other hand, banks’ risk also affects economic activities and price levels. So that riskier conditions on the side of banks’ assets cause adverse selection, and risky investments cause more losses by increasing the probability of default, which will have an effect on variables such as inflation and on the other hand, credit development in an economy can be a factor for increasing financial risk. The unique feature of the presented model is considering the risk of loans to trust enterprises. The model selected in this article is adjusted using the macroeconomic information of Iran’s economy. The results obtained from the simulation of the model show that there is a credit channel and a monetary policy risk channel for the Iranian economy, and i*t* can be said that by applying an expansionary monetary policy shock, the volume of production, inflation, private sector consumption, investment, net worth and the amount of working hours will increase as well as the total amount of loans and the risk of loans. It can also be stated that applying a credit shock through an increase in banks’ lending power will increase production, private sector consumption, investment, net worth and total loans, and inflation will decrease. Also, the application of risk shock will increase inflation and decrease consumption and investment, and the level of production will not change much. It is evident from the obtained results that a significant share of the inflation in recent years in Iran’s economy was caused by the amount of debt in the banking system. By increasing the lending power of the banking system (which can be done through the sale of excess assets and debt collection), it can play a significant role in reducing inflation on the one hand and increasing economic growth on the other hand. For future research directions, the data envelopment analysis (DEA) approach [71–120] can be applied to examine the effect of monetary policy risk channel on operational efficiency of banking industry. Moreover, it is suggested that liquidity risk and market risk can also be considered. Also, in the DSGE model presented in this article, the interbank market and the international connection of different economic sectors have not been considered, which can be discussed in future studies.
